# Endotoxin-Induced Emphysema Exacerbation: A Novel Model of Chronic Obstructive Pulmonary Disease Exacerbations Causing Cardiopulmonary Impairment and Diaphragm Dysfunction

**DOI:** 10.3389/fphys.2019.00664

**Published:** 2019-05-28

**Authors:** Milena Vasconcellos de Oliveira, Nazareth de Novaes Rocha, Raquel Souza Santos, Marcella Rieken Macedo Rocco, Raquel Ferreira de Magalhães, Johnatas Dutra Silva, Sergio Augusto Lopes Souza, Vera Luiza Capelozzi, Paolo Pelosi, Pedro Leme Silva, Patricia Rieken Macedo Rocco

**Affiliations:** ^1^ Laboratory of Pulmonary Investigation, Carlos Chagas Filho Institute of Biophysics, Federal University of Rio de Janeiro, Rio de Janeiro, Brazil; ^2^ Department of Physiology and Pharmacology, Biomedical Institute, Fluminense Federal University, Niterói, Brazil; ^3^ Department of Radiology, Faculty of Medicine, Federal University of Rio de Janeiro, Rio de Janeiro, Brazil; ^4^ Department of Pathology, Faculty of Medicine, University of São Paulo, São Paulo, Brazil; ^5^ Department of Surgical Sciences and Integrated Diagnostics (DISC), University of Genoa, Genoa, Italy; ^6^ San Martino Policlinico Hospital, IRCCS for Oncology and Neurosciences, Genoa, Italy

**Keywords:** emphysema, collagen fiber, lung mechanics, pulmonary arterial hypertension, diaphragm dysfunction

## Abstract

Chronic obstructive pulmonary disease (COPD) is a progressive disorder of the lung parenchyma which also involves extrapulmonary manifestations, such as cardiovascular impairment, diaphragm dysfunction, and frequent exacerbations. The development of animal models is important to elucidate the pathophysiology of COPD exacerbations and enable analysis of possible therapeutic approaches. We aimed to characterize a model of acute emphysema exacerbation and evaluate its consequences on the lung, heart, and diaphragm. Twenty-four Wistar rats were randomly assigned into one of two groups: control (C) or emphysema (ELA). In ELA group, animals received four intratracheal instillations of pancreatic porcine elastase (PPE) at 1-week intervals. The C group received saline under the same protocol. Five weeks after the last instillation, C and ELA animals received saline (SAL) or *E. coli* lipopolysaccharide (LPS) (200 μg in 200 μl) intratracheally. Twenty-four hours after saline or endotoxin administration, arterial blood gases, lung inflammation and morphometry, collagen fiber content, and lung mechanics were analyzed. Echocardiography, diaphragm ultrasonography (US), and computed tomography (CT) of the chest were done. ELA-LPS animals, compared to ELA-SAL, exhibited decreased arterial oxygenation; increases in alveolar collapse (*p* < 0.0001), relative neutrophil counts (*p* = 0.007), levels of cytokine-induced neutrophil chemoattractant-1, interleukin (IL)-1β, tumor necrosis factor-α, IL-6, and vascular endothelial growth factor in lung tissue, collagen fiber deposition in alveolar septa, airways, and pulmonary vessel walls, and dynamic lung elastance (*p* < 0.0001); reduced pulmonary acceleration time/ejection time ratio, (an indirect index of pulmonary arterial hypertension); decreased diaphragm thickening fraction and excursion; and areas of emphysema associated with heterogeneous alveolar opacities on chest CT. In conclusion, we developed a model of endotoxin-induced emphysema exacerbation that affected not only the lungs but also the heart and diaphragm, thus resembling several features of human disease. This model of emphysema should allow preclinical testing of novel therapies with potential for translation into clinical practice.

## Introduction

Chronic obstructive pulmonary disease (COPD) is currently the fourth leading cause of death worldwide and is expected to be the third in 2020 (Lozano et al., 2012). COPD is a disease of the airways and lungs that is characterized by a progressive airflow limitation, which is not fully reversible (Minai et al., 2008). Although essentially a pulmonary disease, it is associated with several extrapulmonary manifestations, such as *cor pulmonale* (Bhatt and Dransfield, 2013), diaphragm dysfunction (Ottenheijm et al., 2008), skeletal muscle wasting (Maltais et al., 2014), and weight loss (Agusti et al., 2003). These systemic manifestations can increase the risk of COPD exacerbations (Cavaillès et al., 2013) and decrease survival (Kawut et al., 2014). Exacerbations, which are defined as an acute worsening of respiratory symptoms that results in additional therapy (Vogelmeier et al., 2017), can be caused or triggered by viral (Seemungal et al., 2000) or bacterial (Stockley et al., 2009) infections. COPD exacerbations result in increased morbidity, hospital admissions, and mortality, and strongly influence health-related quality of life (Wedzicha and Donaldson, 2003). Several experimental models have used lipopolysaccharide (LPS) to induce exacerbation of established emphysema (Hardaker et al., 2010; Ganesan et al., 2012; Kobayashi et al., 2013). Hardaker et al. exposed rats to tobacco smoke for 30 min twice a day for 2 days; on day 3, animals were exposed to LPS for 30 min, followed by exposure to tobacco smoke 5 h later (Hardaker et al., 2010). Ganesan et al. exposed mice to elastase and LPS for four consecutive weeks (Ganesan et al., 2012). Kobayashi et al. developed a model of emphysema with a single dose of elastase followed by intratracheal administration of LPS after 21 days (Kobayashi et al., 2013). The first two studies evaluated lung function and inflammation at day 1, whereas in the latter, animals were evaluated 1, 3, and 7 days after LPS instillation. However, all of these studies focused on pulmonary changes. To our knowledge, no experimental study has focused primarily on the extrapulmonary consequences of emphysema exacerbations. Within this context, the present study aimed to develop a model of endotoxin-induced emphysema exacerbation focused not only on the lungs but also on the heart and diaphragm.

## Materials and Methods

### Ethics Statement

This study was approved by the Ethics Committee of the Health Sciences Center (CEUA-CCS 059-15), Federal University of Rio de Janeiro. All animals received humane care in compliance with the “Principles of Laboratory Animal Care” formulated by the National Society for Medical Research and the *Guide for the Care and Use of Laboratory Animals* prepared by the National Academy of Sciences, USA. The present study followed the ARRIVE guidelines for reporting of animal research (Kilkenny et al., 2010).

### Animal Preparation and Experimental Protocol

**Figure 1:**
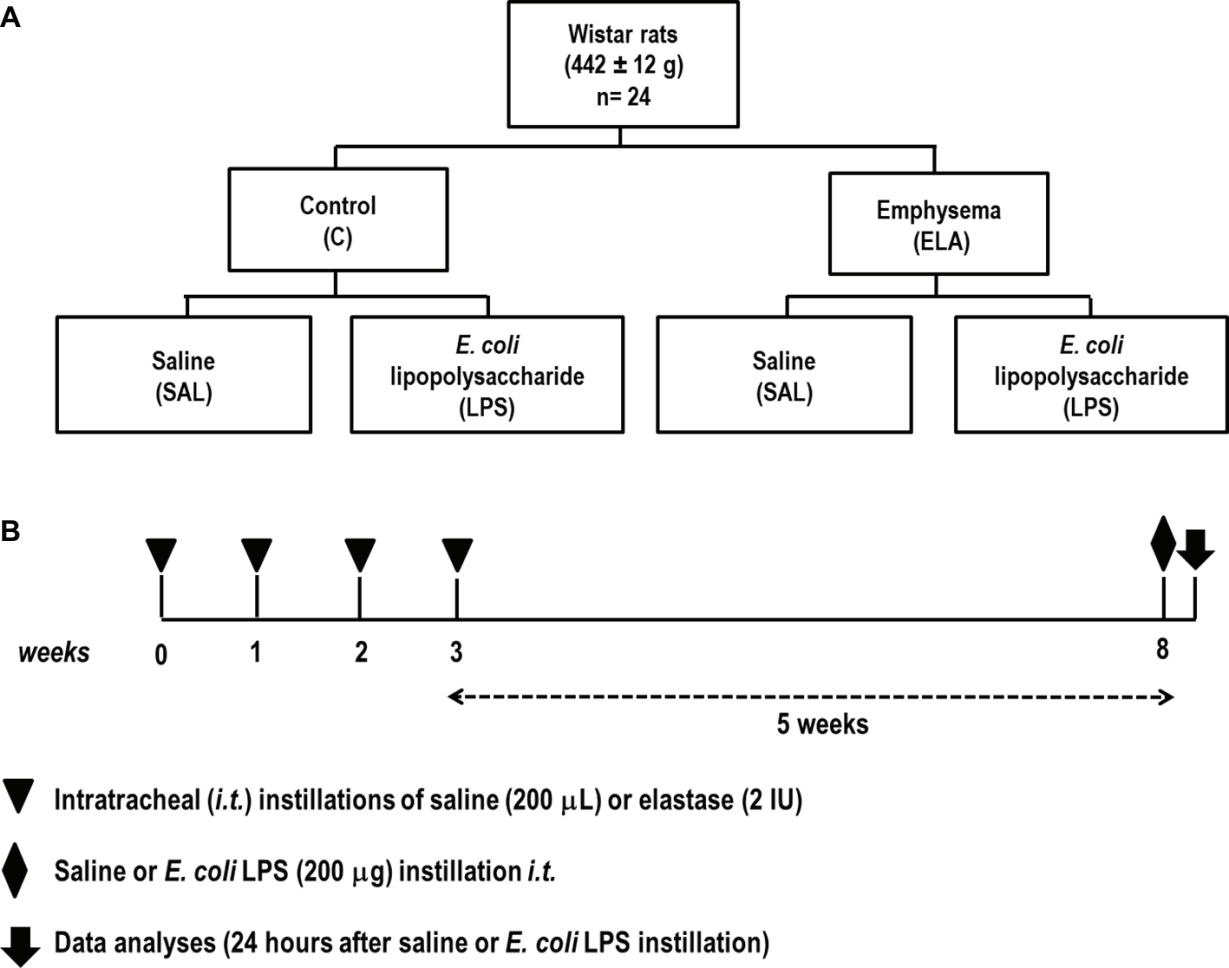
**(A)** Schematic flow chart and **(B)** timeline of the study design. Animals were randomly assigned into two main groups: control (C) and emphysema (ELA). In the ELA group, animals received four intratracheal instillations of pancreatic porcine elastase (PPE) at 1-week intervals. The C group received saline alone using the same protocol. Five weeks after the last instillation, C and ELA animals received saline (SAL) or *E. coli* LPS (LPS) intratracheally.

The time course of interventions is depicted in Figure 1. Twenty-four Wistar rats (weight 442 ± 12 g) were randomly assigned into one of two groups: control (C) and emphysema (ELA). Emphysema was induced according to a protocol previously established by our team (Henriques et al., 2016; Wierzchon et al., 2017). Briefly, animals received four intratracheal instillations of pancreatic porcine elastase (PPE, 2 IU in 0.1 ml of saline solution, Sigma Chemical Co., St. Louis, MO, USA) at 1-week intervals (8 IU PPE in total). The C group received sterile saline (0.1 ml) using the same protocol. Five weeks after the last instillation, C and ELA animals were randomly assigned to receive saline (200 μl, SAL) or *E. coli* lipopolysaccharide (LPS, O55:B5, LPS Ultrapure; InvivoGen, France, 200 μg in 200 μl) intratracheally. Before each intratracheal instillation, animals were anesthetized with 1.5–2.0% isoflurane (Cristália, Itapira, SP, Brazil) by mask.

Twenty-four hours after saline or endotoxin administration, pulmonary function, light and electron microscopy, collagen and elastic fiber content, as well as levels of pro-inflammatory mediators and growth factor in lung-tissue homogenate were analyzed. Echocardiography, diaphragm ultrasonography (US), and CT of the chest were performed.

For functional analysis, animals were anesthetized with diazepam (10 mg/kg intraperitoneally, Compaz®, Cristália, Itapira, SP, Brazil), ketamine (50–100 mg/kg, Ketamin-S+®, Cristália, Itapira, SP, Brazil), and midazolam (2 mg/kg, Dormire®, Cristália, Itapira, SP, Brazil). An intravenous catheter (Jelco 24G) was inserted into the tail vein for continuous infusion of midazolam (2 mg/kg/h), ketamine (50 mg/kg/h), and Ringer’s lactate (8 ml/kg/h, B. Braun, Crissier, Switzerland). Anesthetized animals were tracheotomized *via* a midline neck incision after subcutaneous injection of 2% lidocaine (Xylestesin®, Cristália, Itapira, SP, Brazil). The right internal carotid artery was cannulated (18G, Arrow International, USA) for blood sampling and mean arterial pressure (MAP) measurement. Heart rate (HR), MAP, and rectal temperature were continuously monitored (Networked Multiparameter Veterinary Monitor Life Window 6000V; Digicare Animal Health, Florida, USA). Body temperature was maintained at 37.0 ± 1°C using a heating bed. Animals were mechanically ventilated (Servo-I, MAQUET, Solna, Sweden) in pressure-support mode (PSV) for 5 min with a tidal volume (*V*_T_) of 6 ml/kg, zero end-expiratory pressure, and fraction of inspired oxygen (FiO_2_) set to 0.4. Arterial blood (300 μl) was drawn into a heparinized syringe for measurement of arterial oxygen partial pressure (PaO_2_), arterial carbon dioxide partial pressure (PaCO_2_), arterial pH (pHa), and bicarbonate $\left({\text{HCO}}_{3}^{-}\right)$ (Radiometer ABL80 FLEX, Copenhagen NV, Denmark). Lung mechanics, arterial blood gases, and echocardiographic parameters were measured. At the end of the experiment, heparin (1,000 IU) was injected into the tail vein, a laparotomy was performed, and animals were killed by intravenous injection of sodium thiopental (50 mg/kg, Cristália, Itapira, SP, Brazil). At positive end-expiratory pressure (PEEP) = 3 cmH_2_O, the left and right lungs were removed for histological and molecular biology analysis, respectively.

### Respiratory Data Acquisition and Processing

Airflow was measured using a pneumotachograph (internal diameter = 1.5 mm, length = 4.2 cm, and distance between side ports = 2.1 cm) connected to a SCIREQ differential pressure transducer (UT-PDP-02; SCIREQ, Montreal, Canada). *V*_T_ was calculated by digital integration of the airflow signal. Tracheal pressure (Paw) was measured with a SCIREQ differential pressure transducer (UT-PDP-75; SCIREQ, Montreal, QC, Canada). Esophageal pressure was measured using a 30-cm-long water-filled catheter (PE205) with side holes at the tip connected to a differential pressure transducer (UT-PL-400, SCIREQ, Montreal, Canada) (Baydur et al., 1982). Transpulmonary pressure (*P*_L_) was calculated during inspiration and expiration as the difference between Paw and Pes. Dynamic lung elastance (*E*_L_) was determined by dividing peak transpulmonary pressure (*P*peak_L_) minus total PEEP by delta *V*_T_ (Henriques et al., 2016). Peak (*P*peak_L_) and mean (*P*mean_L_) transpulmonary pressures were calculated. The respiratory rate (RR) was calculated from Pes swings as the frequency per minute of each type of breathing cycle. The ratio between inspiratory and total time (Ti/Ttot), as well as expiratory time (Te), were calculated.

The esophageal pressure (Pes)-time product per minute (PTP/min, integral of ΔPes over time), as well as the esophageal pressure generated 100 ms after onset of inspiratory effort (*P*_0.1_), were calculated. Both measurements reflect work of breathing (Sassoon et al., 1991).

Airflow, Paw, and Pes were continuously recorded throughout the experiments with a computer running software written in LabVIEW® (National Instruments, Austin, Texas, USA) (Samary et al., 2015; Santos et al., 2018). All signals were filtered (200 Hz), amplified by a 4-channel conditioner (SC-24, SCIREQ, Montreal, Quebec, Canada), and sampled at 200 Hz with a 12-bit analog-to digital converter (National Instruments; Austin, Texas, USA). All mechanical data were computed offline by a routine written in MATLAB (Version R2007a; The Mathworks Inc., Natick, Massachusetts, USA).

### Echocardiography

Animals were shaved and placed in the dorsal recumbent position. Transthoracic echocardiography was performed by an expert (NNR) blinded to group allocation, using an UGEO HM70A system (Samsung, São Paulo, Brazil) equipped with a linear phased-array probe (8–13 MHz). Images were obtained from the subcostal and parasternal views. Short-axis two-dimensional views were acquired at the level of the papillary muscles to measure the right ventricular (RV) area, and M-mode images were obtained from the right outflow tract to evaluate the RV wall thickness. The left ventricular ejection fraction (LVEF) was obtained by multiplying the left ventricle (LV) outflow tract area by the velocity–time integral (VTI) on the LV long parasternal view. Pulsed-wave Doppler was used to measure the PAT/PET ratio, an indirect index of pulmonary arterial hypertension (Abbas et al., 2013). HR was assessed from the subcostal view. All parameters were assessed following American Society of Echocardiography and European Association of Cardiovascular Imaging recommendations (Lang et al., 2015).

### Diaphragm Ultrasound

Ultrasonography was performed by the same trained operator (NNR) using an UGEO HM70A system (Samsung, São Paulo, Brazil) equipped with a linear phased-array probe (8–13 MHz). The transducer was angled medially and anteriorly between the right anterior and medial axillary lines so that the ultrasound beam would reach the posterior third of the right hemidiaphragm. The ultrasound was used in B-mode to visualize the diaphragm and then in M-mode to measure the amplitude of craniocaudal diaphragmatic excursion during breathing. Diaphragm thickness was also measured from the most superficial hyperechoic line (pleural line) to the deepest hyperechoic line (peritoneal line). The averaged value of three consecutive measurements was recorded for each. We also calculated the thickening fraction (TF, proportional thickening of the diaphragm), as defined by the following equation (Umbrello et al., 2015):

$$\begin{array}{l} TF\;=\;\mathrm{End}\;\mathrm{inspiratory}\;\mathrm{thickness}\;–\;\mathrm{End}-\mathrm{expiratory}\;\mathrm{thickness}/\\ \;\mathrm{End}-\mathrm{expiratory}\;\mathrm{thickness}\;\times \;100 \end{array}$$

### Lung Histology

#### Light Microscopy

Lung histology analysis was performed by an expert (MVO) blinded by slice coding. Microscopy was performed using an integrating eyepiece with a coherent system consisting of a grid with 100 points and 50 lines of known length coupled to a conventional light microscope (Olympus BX51, Olympus Latin America-Inc., Brazil). The volume fraction of hyperinflated, collapsed, and normal pulmonary areas (× 200 magnification), the mean linear intercept (Lm) (× 400 magnification), and the percentage of mononuclear cells and neutrophils (× 1,000 magnification) in pulmonary tissue were determined by the point-counting technique across 10–20 random, non-overlapping microscopic fields (Hsia et al., 2010). To characterize the heterogeneity of airspace enlargement, a hallmark of emphysema, the central moment of the mean linear intercept (D_2_ of Lm) was computed from 20 measurements (Parameswaran et al., 2006), as per Eq. 1:

$$D_{2}=\mu \cdot \left(1+\frac{\sigma^{2}}{\mu^{2}+\sigma^{2}}\right)\cdot \left(2+\sigma \cdot \frac{\gamma }{\mu }\right)$$ (1)

where *μ* is the mean, *σ* is the variance of airspace diameters, and *γ* is the skewness of the diameter distribution. After *D*_2_ calculation, the heterogeneity index (*β*) was derived from Lm and *D*_2_ values by their ratio (Wierzchon et al., 2017).

The collagen fiber content (Picrosirius-polarization method) was computed in alveolar septa, small airways, and pulmonary vessel wall, while the amount of elastic fiber (Weigert’s resorcin fuchsin method with oxidation) was computed in alveolar septa using Image-Pro Plus 6.0 software (400× magnification) (Fullmer et al., 1974).

#### Transmission Electron Microscopy

##### Lung and Diaphragm Tissue

Three 2 mm × 2 mm × 2 mm slices were cut from three different segments of the right lung and diaphragm and fixed in 2.5% glutaraldehyde and phosphate buffer, 0.1 M (pH 7.4), for electron microscopy analysis (JEOL 1010 Transmission Electron Microscope, Tokyo, Japan). On each lung electron microscopy image (20 fields of view/animal), the following alterations were analyzed: (1) alveolar wall disruption; (2) elastic fiber destruction; (3) neutrophil and macrophage infiltration; (4) detachment of type II epithelial cells (Rocha et al., 2017); (5) endothelial-cell damage (Antunes et al., 2014); and (6) interstitial edema. On each diaphragm electron microscopy image (20 fields/animal), the following changes were analyzed: disorganized I-band glycogen accumulation, thickened Z lines, and mitochondrial aggregates (Padilha et al., 2016). Pathologic findings were graded on a five-point, semiquantitative, severity-based scoring system as follows: 0 = normal lung parenchyma, 1 = changes in 1–25% of examined tissue, 2 = changes in 26–50% of examined tissue, 3 = changes in 51–75% of examined tissue, and 4 = changes in 76–100% of examined tissue of examined tissue (Antunes et al., 2014). The pathologists and technicians working on light microscopy and transmission electron microscopy images were blinded to group assignment.

### Enzyme-Linked Immunosorbent Assay (ELISA)

Levels in lung-tissue homogenate of pro-inflammatory mediators associated with lung inflammation [keratinocyte-derived chemokine, also known as cytokine-induced neutrophil chemoattractant (CINC)-1, a rat analog of interleukin-8; interleukin (IL)-1β; IL-6; and tumor necrosis factor (TNF)-α] and of vascular endothelial growth factor (VEGF, a growth factor which is crucial for pulmonary vessel formation and development) were evaluated by ELISA using matched antibody pairs from PeproTech and R&D Systems (Minneapolis, MN, USA), according to manufacturer instructions. The results were normalized by total protein content by Bradford’s technique, and expressed as picograms per microgram (pg/mg).

### Time-Course Study

To evaluate cardiac and lung structural changes, one additional group of six rats (weight: 436 ± 16 g) was evaluated in the elastase-induced emphysema and endotoxin-induced emphysema exacerbation groups. CT and echocardiography were assessed before (INITIAL) and after (ELA) the emphysema protocol, and after endotoxin-induced emphysema exacerbation (ELA-LPS).

#### Computed Tomography

CT scans were performed to evaluate the presence of emphysematous areas. CT was performed with an Optima 560 positron emission tomography (PET)/CT scanner (GE Healthcare, Boston, USA). The acquisition protocol was based on helical CT with axial slices of 0.625 mm (16 mm × 0.625 mm) thickness and 48 images, with a beam collimation of 10.0 mm and a display field of view of 10 cm. The X-ray tube was set to 120 kV and 80 mA. The total time for each scan was 12 s. Hounsfield units (HU) were analyzed in both lungs, at the bifurcation of pulmonary arteries, in the Onis 2.5 software environment (DigitalCore, Co. Ltd., Tokyo, Japan). All slices were segmented and the CT number in HU was classified as: hyperaerated, from −1,024 to −900 HU; normal aeration, −900 to −500 HU; hypoaerated, −500 to −100 HU; non-aerated, from −100 to 100 HU (Gattinoni et al., 2001). Lung volume was obtained as the sum of each segmented area multiplied by the thickness of the slice. Lung mass was calculated by assuming each voxel was a linear combination of only air and tissue. The specific gravity of lung volume was calculated using the formula (lung HU − air HU)∕(tissue HU − air HU), and mass = specific gravity × total volume × 1.04 g∕cm (Busse et al., 2013).

### Statistical Analysis

The number of animals per group was based on previous studies from our laboratory (Henriques et al., 2016; Wierzchon et al., 2017). A sample size of six animals per group (providing for one animal as dropout) would provide the appropriate power (1 − *β* = 0.8) to identify significant (*α* = 0.05) differences between C and ELA animals, taking into account an effect size *d* = 2.21, a two-sided test, and a sample size ratio = 1 (G^*^Power 3.1.9.2, University of Düsseldorf, Germany).

Data were tested for normality using the Kolmogorov–Smirnov test with Lilliefors’ correction, while the Levene median test was used to evaluate homogeneity of variances. Two-way repeated-measures ANOVA followed by Holm–Šídák’s multiple comparisons was used to compare functional data and histology between groups. Nonparametric data were analyzed using ANOVA on ranks followed by Dunn’s test. Parametric data were expressed as mean ± standard deviation (SD), and nonparametric data, as median (interquartile range). CT and echocardiography changes over time (INITIAL, ELA, and ELA-LPS) were compared by repeated-measures ANOVA followed by Holm–Šídák’s multiple comparisons. All tests were performed in GraphPad Prism v6.07 (GraphPad Software, La Jolla, California, USA).

## Results

The survival rate was 100% in all groups.

### Pulmonary Effects

#### Effects of Endotoxin in Control Animals

**Figure 2:**
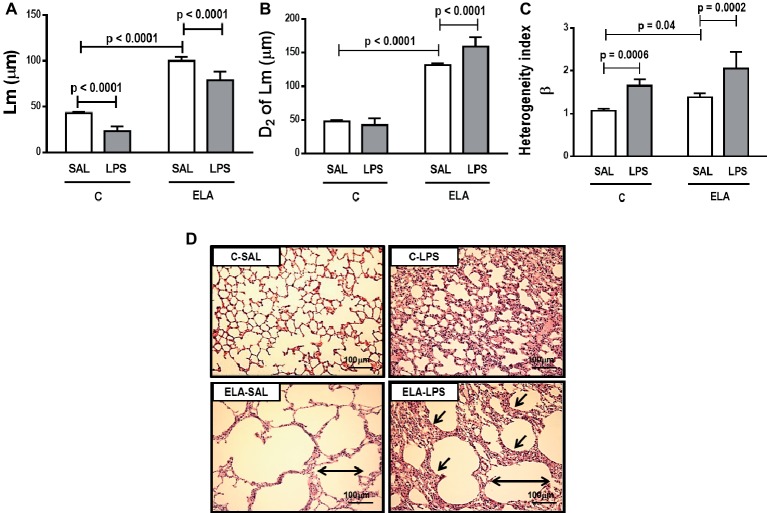
**(A)** Mean linear intercept (Lm). **(B)** Central moments of mean linear intercept (D2 of Lm). **(C)** Heterogeneity index (β) and **(D)** representative photomicrographs of lung parenchyma stained with hematoxylin-eosin (HE) (400× magnification). Note alveolar space enlargement in the ELA groups (horizontal arrows) and increased alveolar collapse after exacerbation (sloping arrows) (ELA-LPS group). C, control; ELA, emphysema; SAL, animals treated with saline; LPS, animals treated with *E. coli* LPS. Values are means + SD of six animals in each group.

**Figure 3:**
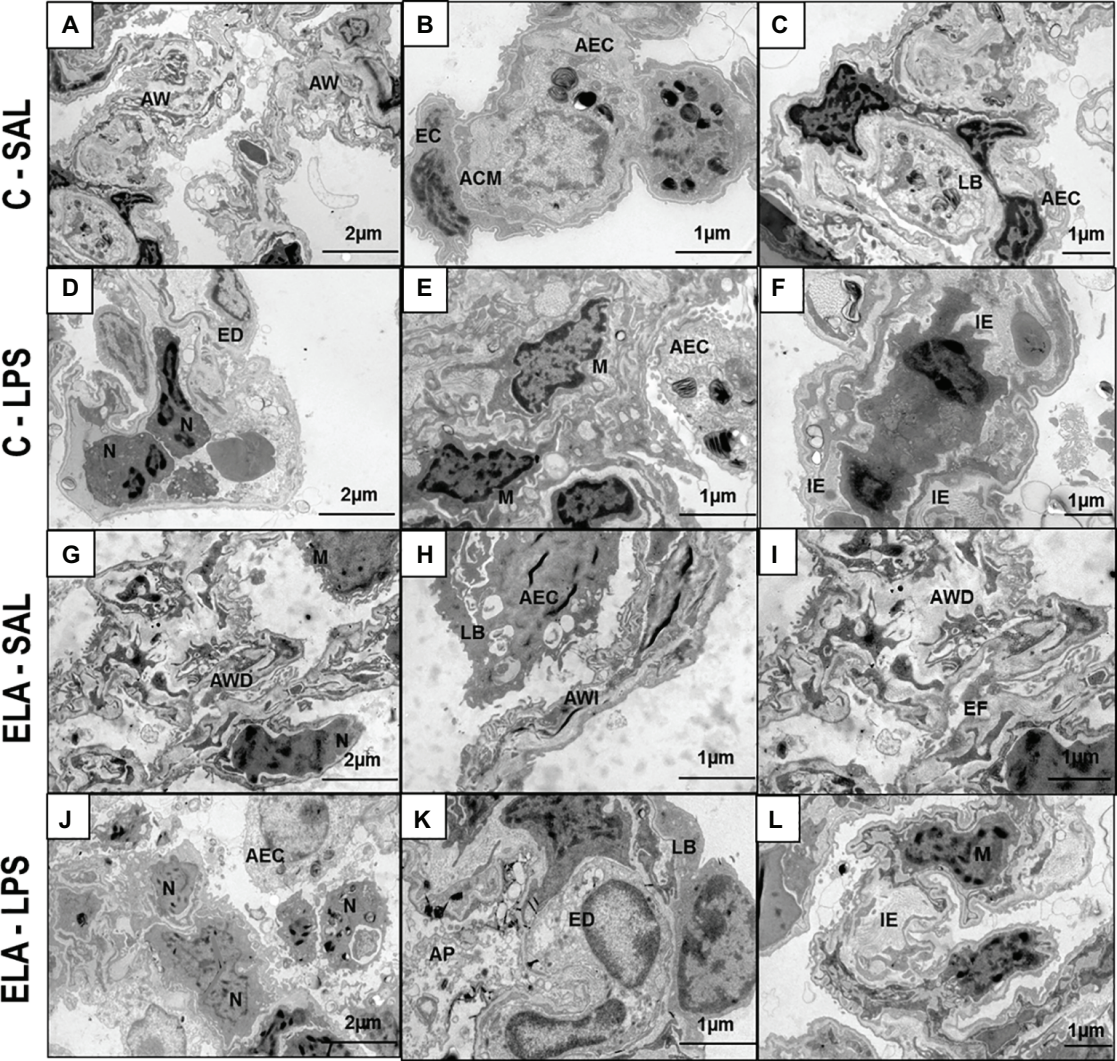
Transmission electron microscopy of lung parenchyma in control (C) and animals exposed to saline **(A–C)** or *E. coli* LPS **(D–F)**, as well as in emphysema (ELA) rats exposed to saline **(G–I)** or *E. coli* LPS **(J–L)**. Note preserved alveolar walls (AW), integrity of endothelial cells (EC) of the alveolar–capillary membrane (ACM), and lamellar bodies (LB) of alveolar epithelial cells (AEC) in the C-SAL group. C-LPS animals show collapse of AW with increased neutrophil (N) and macrophage (M) counts, endothelial-cell damage (ED), detachment of AEC with increased permeability of the alveolar–capillary membrane, and interstitial edema (IE). ELA-SAL animals show alveolar wall disruption (AWD) and irregularity (AWI), inflammation, and destruction of elastic fibers (EF). The pattern of inflammation involves increased numbers of neutrophils (N) and macrophages (M). These cells produce proteases involved in tissue destruction and release local and systemic inflammatory mediators, which amplify inflammation and structural disruption. Note detachment of AEC and reduction in LB. After emphysema exacerbation, there is an increase in neutrophil (N) and macrophage (M) response, increased detachment of AEC, loss of LB, and ED, causing increased permeability of the alveolar–capillary membrane and IE due to surfactant abnormalities.

**Figure 4:**
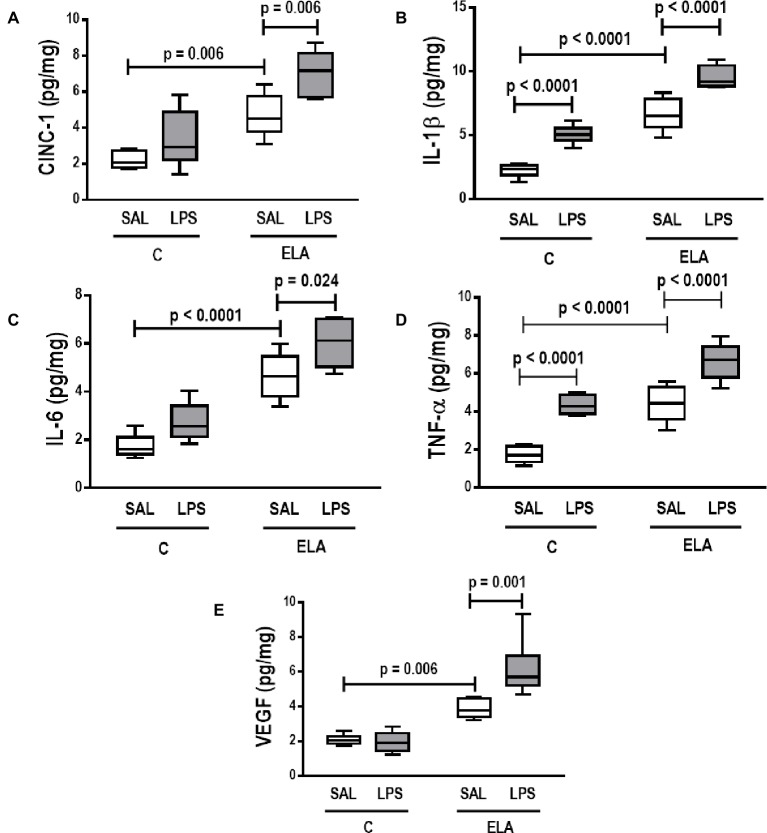
Levels of **(A)** keratinocyte-derived chemokine (cytokine-induced neutrophil chemoattractant [CINC]-1, a rat analog of interleukin-8), **(B)** interleukin (IL)-1β, **(C)** IL-6, **(D)** tumor necrosis factor (TNF)-α, and **(E)** vascular endothelial growth factor (VEGF) in lung tissue. C, control; ELA, animals treated with intratracheal instillations of elastase; SAL, saline; LPS, *E. coli* lipopolysaccharide. Values are median (interquartile range) of six animals in each group.

**Figure 5:**
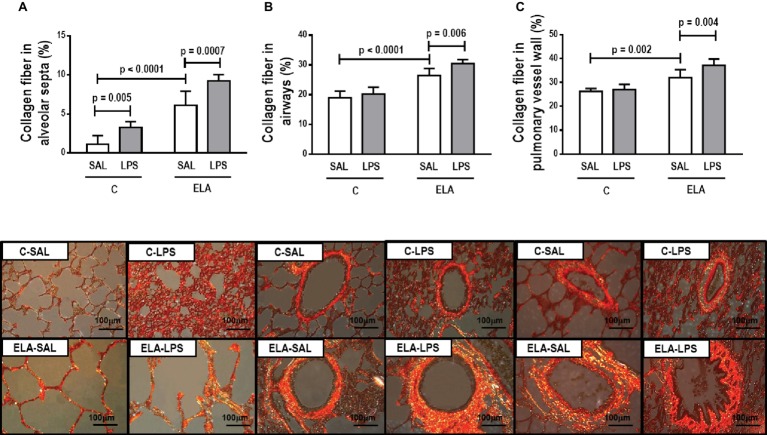
Collagen fiber content and representative photomicrographs of **(A)** alveolar septa, **(B)** airways, and **(C)** pulmonary vessel wall stained with the Picrosirius-polarization method (collagen fibers). C, control; ELA, animals treated with intratracheal instillations of elastase; SAL, saline; LPS, *E. coli* lipopolysaccharide. Values are mean ± SD of six animals in each group.

**Figure 6:**
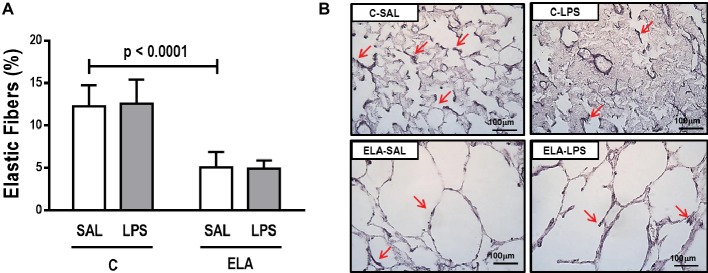
**(A)** Elastic fiber content in alveolar septa and **(B)** representative photomicrographs of the lung parenchyma stained with Weigert’s resorcin fuchsin method with oxidation (elastic fibers). Red arrows: elastic fibers are stained in black. C, control; ELA, animals treated with intratracheal instillations of elastase; SAL, saline; LPS, *E. coli* lipopolysaccharide. Values are mean ± SD of six animals in each group.

C-LPS animals, compared to C-SAL, showed increased *E*_L_ (Table 1), alveolar collapse (31-fold increase), neutrophil infiltration (4.4-fold increase) (Supplementary Figure S1; Table 2), and heterogeneity index β (Figure 2), as well as reduced PaO_2_/FiO_2_ (Table 1) and Lm (Figure 2). Ultrastructural analyses of lung parenchyma exhibited increased neutrophil and macrophage infiltration, detachment of type II epithelial cells, endothelial-cell damage, and interstitial edema in C-LPS rats (Table 3; Figure 3). Levels of TNF-α and IL-1β (Figure 4), as well as collagen fiber content in alveolar septa (3-fold increase) (Figure 5), were higher in C-LPS than C-SAL, while elastic fiber content did not differ between groups (Figure 6).

**Table 1:** Respiratory mechanics and arterial blood gases.

	C		ELA	
	SAL	LPS	SAL	LPS
***Spontaneous breathing***
*V*_T_ (ml/kg)	6.0 ± 0.1	5.8 ± 0.3	5.7 ± 0.4	5.8 ± 0.4
RR (breaths/min)	41 ± 4	44 ± 4	46 ± 7	50 ± 7
Ti (s)	0.55 ± 0.07	0.47 ± 0.06	0.33 ± 0.08^*^	0.31 ± 0.08
Te (s)	0.84 ± 0.06	0.86 ± 0.05	1.10 ± 0.11^*^	0.92 ± 0.16
Ti/Ttot (s)	0.38 ± 0.03	0.35 ± 0.02	0.26 ± 0.05^*^	0.28 ± 0.05
*E*_L_ (cmH_2_O/ml)	3.2 ± 0.2	4.3 ± 0.5^*^	4.9 ± 0.6^*^	6.8 ± 0.4^#^
PTP/min (cmH_2_O.s/min)	11.5 ± 2.2	12.8 ± 1.3	19.4 ± 2.8^*^	27.1 ± 5.5^#^
*P*_0.1_ (cmH_2_O)	−1.04 ± 0.44	−1.07 ± 0.57	−1.93 ± 0.53	−2.33 ± 0.67
***Arterial blood gases***
PHa	7.39 ± 0.06	7.38 ± 0.06	7.36 ± 0.08	7.33 ± 0.06
PaCO_2_ (mmHg)	38 ± 3	40 ± 4	42 ± 5	44 ± 5
PaO_2_/FiO_2_ (mmHg)	428 ± 24	295 ± 48^*^	349 ± 41	272 ± 59^#^
HCO_3_ (mmol/L)	22 ± 1	21 ± 1	24 ± 1	24 ± 2

**V*_T_, tidal volume; RR, respiratory rate; Ti, inspiratory time; Te, expiratory time; Ti/Ttot, inspiratory time divided by total respiratory cycle time; *E*_L_, dynamic lung elastance; PTP/min pressure–time product per minute; *P*_0.1_ esophageal pressure generated 100 ms after onset of inspiratory effort; pHa, arterial pH; PaCO_2_, arterial partial pressure of carbon dioxide; PaO_2_/FiO_2_, arterial partial pressure of oxygen divided by fraction of inspired oxygen (0.21); HCO_3_, bicarbonate. C, control; ELA, animals treated with intratracheal instillations of elastase; SAL, saline; LPS, *E. coli* lipopolysaccharide. Values are means ± SD of six animals in each group*.

* *Significantly different from C-SAL group (*p* < 0.05)*.

# *Significantly different from ELA-SAL group (*p* < 0.05)*.

**Table 2:** Lung morphometry.

	C		ELA	
	SAL	LPS	SAL	LPS
Normal (%)	98.5 ± 0.5	56.7 ± 3.3^*^	52.3 ± 2.9^*^	38.9 ± 1.3^#^
Hyperinflation (%)	0.0 ± 0.0	0.0 ± 0.0	37.9 ± 2.6	39.7 ± 1.0
Collapse (%)	1.4 ± 0.5	43.2 ± 3.3^*^	9.7 ± 0.9^*^	21.3 ± 1.4^†^ ^,^ ^#^
Mononuclear cells (%)	31.3 ± 1.7	50.5 ± 1.8^*^	43.4 ± 1.1^*^	55.0 ± 1.8^#^
Neutrophils (%)	2.0 ± 0.8	8.8 ± 1.4^*^	6.9 ± 1.1^*^	9.9 ± 1.6^#^

*Fractional area of normal alveoli, lung hyperinflation, collapsed alveoli, mononuclear cells, and neutrophils in lung tissue. C, control; ELA, animals treated with intratracheal instillations of elastase; SAL, saline; LPS, *E. coli* lipopolysaccharide. Values are means ± SD of six animals in each group*.

* *Significantly different from C-SAL group (*p* < 0.05)*.

† *Significantly different from C-LPS group (*p* < 0.05)*.

# *Significantly different from ELA-SAL group (*p* < 0.05)*.

**Table 3:** Semiquantitative analysis of lung electron microscopy.

	C		ELA	
	SAL	LPS	SAL	LPS
Alveolar wall destruction	0 (0–0.25)	0.5 (0–1)	4 (3.75–4)^*^	3.5 (3–4)^†^
Elastic fiber rupture	0 (0–0)	0 (0–0)	4 (3–4)	3 (3–4)
Neutrophil infiltration	1 (1–1.25)	2 (2–2.5)^*^	2 (2–2)^*^	4 (3.75–4)^†^ ^,^ ^#^
Macrophage infiltration	1.5 (1–2)	3 (2.75–3)^*^	2.5 (2–3)^*^	4 (4–4)^†^ ^,^ ^#^
Detachment of type II epithelial cells	0 (0–1)	2 (2–3)^*^	1.5 (1–2)^*^	4 (3.75–4)^†^ ^,^ ^#^
Endothelial-cell damage	0 (0–0)	2.5 (2–3)	1.5 (1–2)	4 (3–4)^†^ ^,^ ^#^
Interstitial edema	0 (0–0.25)	3 (2–3)^*^	1 (1–1.25)^*^	4 (4–4)^†^ ^,^ ^#^

*Pathologic findings were graded on a five-point, semiquantitative, severity-based scoring system, where 0, normal lung parenchyma; 1, changes in 1–25% of examined tissue; 2, changes in 26–50% of examined tissue; 3, changes in 51–75% of examined tissue; and 4, changes in 76–100% of examined tissue. C, control; ELA, animals treated with intratracheal instillations of elastase; SAL, saline; LPS, *E. coli* lipopolysaccharide. Analyses performed using ANOVA on ranks followed by Dunn’s test. Values are median (interquartile range) of six animals in each group*.

* *Significantly different from C-SAL group (*p* < 0.05)*.

† *Significantly different from C-LPS group (*p* < 0.05)*.

# *Significantly different from ELA-SAL group (*p* < 0.05)*.

#### Emphysema Induced by Multiple Elastase Instillations

ELA-SAL animals, compared to C-SAL, exhibited increased PTP/min, *E*_L_, Te (Table 1), Lm, *D*_2_ of Lm, heterogeneity index β (Figure 2), neutrophil infiltration (3.5-fold increase), as well as fraction area of alveolar collapse (7-fold increase) and hyperinflation (Supplementary Figure S1; Table 2), as well as decreased Ti and Ti/Ttot (Table 1). Electron microscopy analysis demonstrated alveolar wall destruction, elastic fiber rupture, neutrophil and macrophage infiltration, detachment of type II epithelial cells, endothelial-cell damage, and interstitial edema in ELA-SAL animals (Table 3; Figure 3). Moreover, in ELA-SAL, CINC-1, IL-1β, IL-6, TNF-α, and VEGF levels in lung-tissue homogenate (Figure 4) were increased. ELA-SAL animals presented a modest increase in collagen fiber content in airways (1.4-fold increase) and pulmonary vessel wall (1.2-fold increase) (Figure 5), and a 5.5-fold increase in collagen fiber content in alveolar septa. Elastic fiber content was reduced in ELA-SAL compared to C-SAL (2.4-fold decrease) (Figure 6).

#### Endotoxin-Induced Emphysema Exacerbation

ELA-LPS animals, compared to ELA-SAL, showed further increases in PTP/min, *E*_L_, (Table 1), fraction area of alveolar collapse (2.2-fold increase), percentage of neutrophils (1.5-fold increase) (Table 2), *D*_2_ of Lm, and heterogeneity index β (Figure 2), as well as decreased PaO_2_/FiO_2_ (Table 1) and Lm (Figure 2). ELA-LPS animals exhibited more evident neutrophil and macrophage infiltration, detachment of type II epithelial cells, endothelial-cell damage, and interstitial edema compared to ELA-SAL (Table 3; Figure 3). In ELA-LPS, CINC-1, IL-1β, IL-6, TNF-α, and VEGF levels in lung-tissue homogenate were increased even further compared to ELA-SAL (Figure 4). The amount of collagen fibers in alveolar septa (3-fold increase), airways (1.5-fold increase), and pulmonary vessel walls (1.4-fold increase) was higher in ELA-LPS than ELA-SAL (Figure 5), but no significant changes were observed in elastic fiber content between groups (Figure 6).

*P*_0.1_, *V*_T_, PaCO_2_, RR, pHa, and HCO_3_ did not differ between groups (Table 1).

### Extrapulmonary Effects

#### Cardiac Function

**Figure 7:**
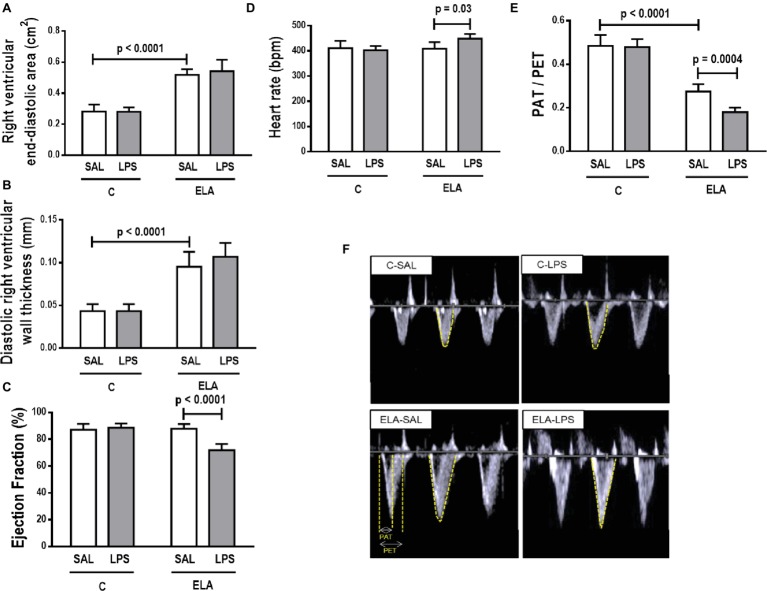
Echocardiography. **(A)** right ventricular end-diastolic area, **(B)** diastolic right ventricular wall thickness, **(C)** ejection fraction, **(D)** heart rate, **(E)** PAT/PET ratio, and **(F)** representative images of pulmonary blood flow. C, control; ELA, animals treated with intratracheal instillations of elastase; SAL, saline; LPS, *E. coli* lipopolysaccharide. Values are mean ± SD of six animals in each group.

Cardiac function was evaluated using echocardiography. The parameters analyzed did not differ between C-SAL and C-LPS (Figure 7). In ELA-SAL, compared to C-SAL, RV end-diastolic area and diastolic RV wall thickness were higher, while PAT/PET was lower (1.8-fold decrease). In ELA-LPS, compared to ELA-SAL, PAT/PET (2.7 fold decrease) and ejection fraction were reduced, whereas HR was increased.

#### Diaphragm Function and Morphology

**Figure 8:**
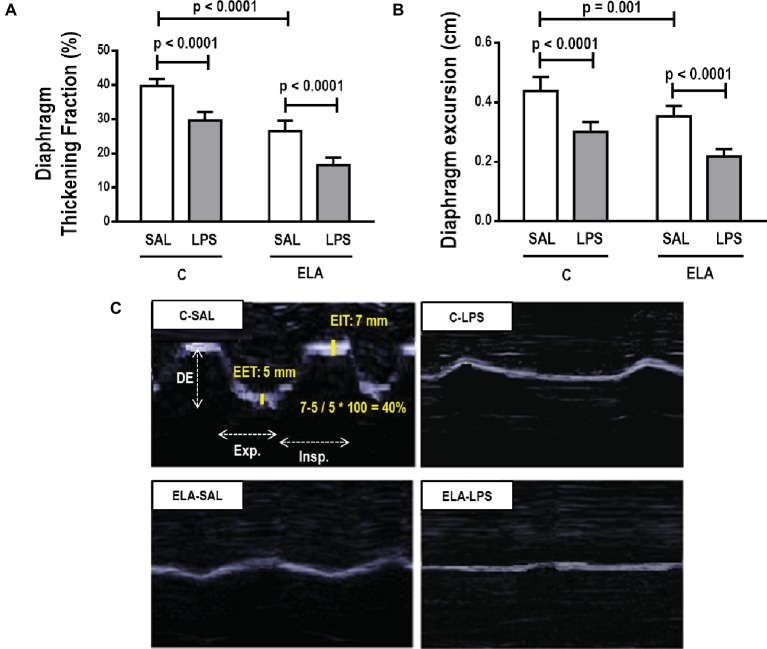
Diaphragm ultrasound. **(A)** diaphragm thickening fraction, **(B)** diaphragm excursion, and **(C)** ultrasonographic view of diaphragmatic excursion (DE) and its thickening fraction (TF), calculated by end inspiratory thickness (EIT) – end-expiratory thickness (EET)/end-expiratory thickness × 100. C, control; ELA, animals treated with intratracheal instillations of elastase; SAL, saline; LPS, *E. coli* lipopolysaccharide. Values are mean ± SD of six animals in each group.

Diaphragm TF and excursion were significantly reduced in C-LPS compared to C-SAL, ELA-SAL compared to C-SAL, and ELA-LPS compared to ELA-SAL (Figure 8). Ultrastructural analysis of the diaphragm showed no modifications in C-LPS compared to C-SAL animals (Table 4). Disorganized I-band, thickened Z lines, mitochondrial subsarcolemmal aggregates, and distortion of mitochondrial cristae were greater in ELA-SAL compared to C-SAL (Table 4; Figure 9), and were even worse in ELA-LPS, compared to ELA-SAL.

**Table 4:** Semiquantitative analysis of diaphragm electron microscopy.

	C		ELA	
	SAL	LPS	SAL	LPS
Disorganized I-band	1 (0.50–1)	1 (1–1.75)	4 (3–4)^*^	3.5 (3–4)^†^
Thickened Z lines	1 (1–1.5)	1.5 (1–2)	3 (2–3)^*^	4 (4–4)^†^ ^,^ ^#^
Mitochondrial subsarcolemmal aggregates	1 (1–1.5)	2 (1.25–2)	3 (2.5–3)^*^	4 (4–4)^†^ ^,^ ^#^
Mitochondrial cristae distortion	0 (0–1.5)	1.5 (1–2)	3 (3–4)^*^	4 (3.25–4)^†^ ^,^ ^#^
Abnormal mitochondrial forms	0 (0–1)	1.5 (1–2)	2 (2–2.5)^*^	4 (3.25–4)^†^ ^,^ ^#^

*Pathologic findings were graded on a five-point, semiquantitative, severity-based scoring system, where 0, normal diaphragm tissue; 1, changes in 1–25% of examined tissue; 2, changes in 26–50% of examined tissue; 3, changes in 51–75% of examined tissue; and 4 = changes in 76–100% of examined tissue. C, control; ELA, animals treated with intratracheal instillations of elastase; SAL, saline; LPS, *E. coli* lipopolysaccharide. Analyses performed using ANOVA on ranks followed by Dunn’s test. Values are median (interquartile range) of six animals in each group*.

* *Significantly different from C-SAL group (*p* < 0.05)*.

† *Significantly different from C-LPS group (*p* < 0.05)*.

# *Significantly different from ELA-SAL group (*p* < 0.05)*.

**Figure 9:**
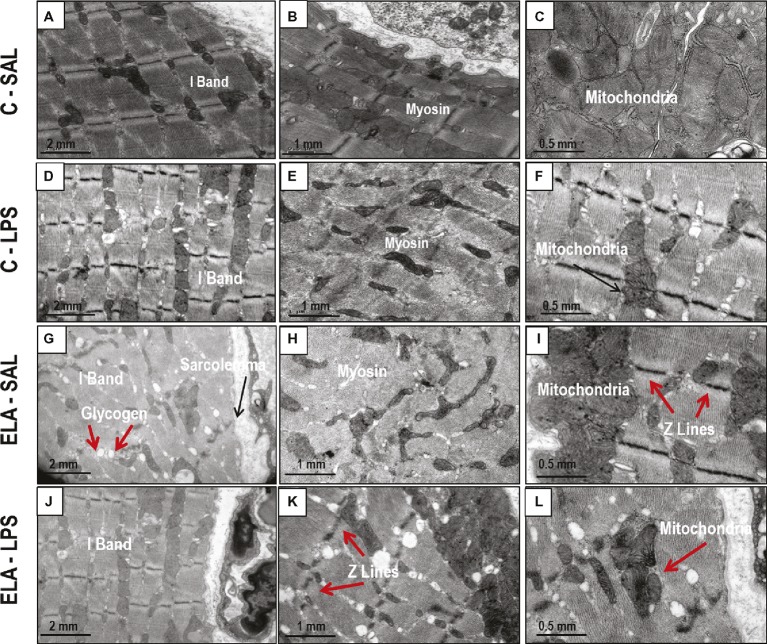
Transmission electron microscopy of diaphragm in control (C) animals treated with saline **(A–C)** or *E. coli* LPS **(D–F)**, as well as in emphysema (ELA) animals treated with saline **(G–I)** or *E. coli LPS* **(J–L)**. Note I-band integrity and preserved myosin and mitochondria in C-SAL group. The C-LPS group shows discrete changes in these structures. The ELA-SAL group exhibits disorganized I-bands, glycogen accumulation in sarcoplasm, loss of myosin content in myofibrils, subsarcolemmal mitochondrial aggregates, and thickened Z lines. The ELA-LPS group shows even more intense changes in these structures.

#### Time-Course Evaluation of Chest CT and Echocardiography Findings

CT showed that animals at the INITIAL condition exhibited normal lung parenchymal densities, ranging from −695 to −514 HU. After induction of emphysema (ELA) and after exacerbation (ELA-LPS), densities increased, ranging from −985 to −815 HU and −867 to −699 HU, respectively. Specific gravity was also higher in ELA compared to INITIAL, and increased further after emphysema exacerbation. There were no changes in lung volume after exacerbation (Supplementary Figure S2).

Echocardiography showed increased RV end-diastolic area, increased diastolic RV wall thickness, and reduced PAT/PET ratio in ELA compared to INITIAL. PAT/PET ratio was lower than in ELA-LPS compared to ELA (Supplementary Figure S3).

## Discussion

In the present experimental study, endotoxin-induced emphysema exacerbation promoted changes in the lung, heart, and diaphragm. In the lungs, emphysema exacerbation resulted in: (1) increased alveolar collapse and airspace heterogeneity; (2) detachment of alveolar type II epithelial cells, endothelial-cell damage, and interstitial edema; (3) infiltration of neutrophils and macrophages in lung tissue; (4) greater deposition of collagen fibers in alveolar septa, airways, and pulmonary vessel walls; (5) impairment of arterial oxygenation; and (6) increased dynamic lung elastance and work of breathing. In the heart, emphysema exacerbation reduced LVEF, increased HR, and reduced PAT/PET ratio, suggesting worsening of pulmonary arterial hypertension. Finally, in the diaphragm, emphysema exacerbation led to disorganized I-bands, thickened Z lines, mitochondrial subsarcolemmal aggregates, and distortion of mitochondrial cristae, as well as reduced diaphragm TF and excursion. A time-course imaging study confirmed that exacerbation caused cardiorespiratory impairment, as demonstrated by an increased percentage of low-attenuation areas and specific gravity on CT and reduced PAT/PET ratio. The strength of the present study is that it is the first to investigate not only lung function and morphological changes, but also the progression of cardiac and diaphragm dysfunction over 24 h in a model of endotoxin-induced exacerbation of elastase-induced emphysema.

The elastase-induced emphysema model and exacerbation caused by LPS instillation.

In the experimental model used herein, emphysema was induced by multiple instillations of PPE (Henriques et al., 2016; Padilha et al., 2016; Wierzchon et al., 2017). This model does not employ the primary disease-causing agent of COPD in humans, which is cigarette smoke. However, smoke-exposure models are difficult to set up, require prolonged exposure (Churg and Wright, 2007; Antunes and Rocco, 2011), and do not induce significant emphysematous changes or pulmonary function abnormalities consistent with advanced disease (Jobse et al., 2014). Protease dysregulation can also cause COPD in humans (e.g., in patients with *α* − 1 antitrypsin deficiency), thereby providing a further rationale for the use of elastase to induce features of COPD in rats (Abboud and Vimalanathan, 2008). Moreover, multiple elastase administrations can lead to extrapulmonary effects that closely resemble those seen in human emphysema (Smith and Wrobel, 2014), such as cor pulmonale (Antunes et al., 2014; Oliveira et al., 2016), cachexia (Rocha et al., 2017), diaphragmatic dysfunction, and exercise intolerance (Lüthje et al., 2009).

The majority of acute exacerbations in COPD patients are caused by Gram-negative bacteria (Albertson and Chan, 2009), which colonize the airways even when disease is stable (Tufvesson et al., 2017). LPS constitutes the major part of the outer membrane of Gram-negative bacteria (Erridge et al., 2002), and recent studies from our group have shown that intratracheal administration of LPS can cause lung damage, characterized by impaired lung mechanics, atelectasis, damage to epithelium and alveolar–capillary membrane, and interstitial edema in healthy rats within 24 h (Riva et al., 2008; Samary et al., 2015; Santos et al., 2018). Endotoxin has been used to exacerbate elastase-induced emphysema models, resulting in bronchial mucus cell hyperplasia (Stolk et al., 1992), neutrophil infiltration, and irreversible alveolar destruction (Kobayashi et al., 2013). However, unlike in these previous studies, we amplified our readouts by evaluating extrapulmonary effects, such as cardiovascular and diaphragmatic complications, which are often the cause of death in patients with emphysema (Chaouat et al., 2008; Barreiro and Gea, 2015; Axson et al., 2018).

### Pulmonary Changes After Emphysema Exacerbation

The emphysema model used herein led to greater areas of lung hyperinflation coexisting with alveolar collapse, which is in line with increased heterogeneity of airspace enlargement (Wierzchon et al., 2017), thus resulting in increased dynamic lung elastance. After emphysema exacerbation, hyperinflation did not change, while alveolar collapse increased; this, in turn, reduced Lm while increasing airspace heterogeneity, yielding a further increase in dynamic lung elastance. Our data stand in contrast to those of a model of emphysema exacerbation in which Lm was found to increase after LPS instillation in mice previously treated with elastase (Kobayashi et al., 2013). There are essentially two explanations for these differences: first, the elastase and LPS dosages used herein were two-fold and eight-fold higher, respectively, than those used by Kobayashi et al.; second, rats have larger alveoli (average 100 μm of Lm) than mice (80 μm) at baseline conditions (Irvin and Bates, 2003). Thus, the rats used in the present study already had a higher Lm with significant airspace heterogeneity (Henriques et al., 2016; Padilha et al., 2016; Wierzchon et al., 2017); LPS instillation led to even greater inflammatory response and alveolar collapse. Sajjan et al. exposed animals to elastase through the intranasal route on day 1 and *E. coli* LPS on day 4 of the week for four consecutive weeks. Lung elastance reduced in both elastase and elastase/LPS groups, with no changes in the LPS group compared with PBS. Conversely, in our study, *E*_L_ was increased after endotoxin-induced exacerbation. This difference is attributable not only to study design, but also to the route of administration (intratracheal versus intranasal instillation) and the dosage of both elastase and LPS.

Elastolysis, a hallmark of emphysema (Antunes et al., 2014; Padilha et al., 2015; Oliveira et al., 2016), did not change after exacerbation. Nevertheless, we observed a more evident detachment of type II epithelial cells and endothelial-cell damage, which also explains the increase in collapsed areas, likely due to surfactant reduction (Ketko and Donn, 2014) and increased interstitial edema, respectively. Lung inflammation increased after emphysema induction and LPS instillations, which corresponds to a “two-hit” model of injury. After priming the lungs by induction of emphysema (“first hit”), LPS administration further increased lung inflammation, as shown by the increased percentage of mononuclear cells and neutrophils in lung tissue (“second hit”). These cells release pro-inflammatory cytokines, such as IL-1β, IL-6, TNF-α, and CINC-1, contributing to disease progression (Barnes, 2008). In this line, IL-1β markedly activates macrophages to secrete inflammatory cytokines, chemokines, and matrix metalloproteinases (Culpitt et al., 2003). IL-6 is involved in systemic features of COPD, such as muscle weakness and endothelial dysfunction (Bhowmik et al., 2000), while TNF-α is usually associated with airway inflammation through activation of the nuclear factor kappa B pathway (Kips et al., 1993). CINC-1 attracts neutrophils and is correlated with an increased proportion of these cells in the sputum of emphysema patients (Traves et al., 2002). VEGF levels are known to decline after emphysema induction, which suggests endothelial-cell injury (Kasahara et al., 2001; Cruz et al., 2012; Girón-Martínez et al., 2014). Nevertheless, in the present study, VEGF levels were higher after emphysema induction and increased further after LPS instillations. We hypothesized that, after emphysema exacerbation, lung endothelial cells did indeed sustain more damage, while the remaining undamaged and functional endothelial cells were able to increase VEGF levels. A similar finding was reported in a previous study by our group (Oliveira et al., 2016).

As a result of the inflammatory process, new collagen fibers are resynthesized as an attempt to maintain lung structure (Suki et al., 2003). In the present study, emphysema development increased collagen fiber deposition in the alveolar septa, airways, and pulmonary vessel walls, which is consistent with previous studies (Cruz et al., 2012; Antunes et al., 2014; Oliveira et al., 2016; Rocha et al., 2017). The amount of collagen fibers in the alveolar septa was also increased 24 h after LPS administration in control rats (Araújo et al., 2010; Maron-Gutierrez et al., 2013; Silva et al., 2018). By contrast, a recent study did not show increased collagen deposition 24 h after nebulized LPS administration (Costa et al., 2017), likely due to the dosage and technique of intratracheal instillation of endotoxin. After endotoxin-induced exacerbation of established emphysema, the amount of collagen fibers was increased, due to higher proliferation of inflammatory cells and cytokines, which worsened the remodeling process.

Both dynamic lung elastance and PTP/min increased after emphysema exacerbation. The former can be explained by maximal hyperinflation and fibrosis in the alveoli, which limited the distension capacity of the lung and increased its elastic recoil (Henriques et al., 2016). PTP/min is a surrogate of work of breathing, suggesting increased respiratory effort due to increased alveolar collapse, lung inflammation, and reduced PaO_2_/FiO_2_ ratio. This scenario has been observed in hospitalized patients (Umbrello et al., 2015). Additionally, Ti/Ttot has been considered an important variable associated with dynamic hyperinflation in emphysema (Marini, 2011). In this line, Ti reduced while Te increased, which suggests expiratory flow limitation due to airflow obstruction (Baldi et al., 2001). After exacerbation, Ti/Ttot did not change further; this probably reflects the restrictive conditions induced by LPS administration.

### Extrapulmonary Consequences of Emphysema Exacerbation

PAT/PET ratio, an indirect index of pulmonary arterial hypertension, was reduced after emphysema development. This can be explained by increased collagen fiber content in lung vessels, which may reduce the diameter of the vascular lumen (Schreier et al., 2013; Oliveira et al., 2016; Rocha et al., 2017). This causes increased afterload and, consequently, induces morphological changes in the right ventricle, such as increased area and wall thickness (Oliveira et al., 2016). The worsening of pulmonary arterial hypertension observed after emphysema exacerbation can be explained by hypoxemia (Siebenmann and Lundby, 2015). Structural changes in the right ventricle were not observed, probably due to the short time elapsed between exacerbation induction and analysis (De Souza et al., 2017). Nevertheless, a reduction in LVEF was noticeable, which may be associated with increased left ventricular end-diastolic volume (which, in turn, reflects impairment of contractility). HR increased after emphysema exacerbation, which represents an attempt to preserve cardiac output.

Diaphragmatic dysfunction is a consequence of emphysema development that can lead to respiratory failure (Unal et al., 2000; Ottenheijm et al., 2007; Baria et al., 2014; He et al., 2014). Emphysema promotes higher lung volumes (Newell, 2008), as observed by CT images in the time-course study. This leads to flattening of the diaphragm, which causes mechanical disadvantage during the respiratory cycle (Similowski et al., 1991). The diaphragmatic functional impairment observed after exacerbation, as depicted in our study by changes in TF and excursion, may be explained by several factors, including greater recruitment of inflammatory cells into the lungs (Gan et al., 2004;Meduri et al., 2009), maintenance of hyperinflated areas (Ottenheijm et al., 2007), and changes in mitochondrial structure.

Dysregulated mitochondrial dynamics play an important role in the development of diaphragmatic weakness and dysfunction (Picard et al., 2015). Additionally, it is well established that the imbalance between fission and fusion can change mitochondrial morphology dramatically (Picard et al., 2015). We observed subsarcolemmal mitochondrial aggregates in the diaphragm after emphysema development and exacerbation, which may result in diaphragmatic dysfunction (Westermann, 2010; Nunnari and Suomalainen, 2012). Disorganized sarcomere I-bands and thickened Z lines were also observed, which suggest contractile damage (Padilha et al., 2016).

CT is one of the most accurate imaging techniques for detection of pulmonary emphysema *in vivo* (Thurlbeck and Müller, 1994). Objective quantitation of emphysema can be accomplished by measuring the relative lung area occupied by pixels with attenuation coefficients (CT numbers) below a predetermined threshold (Sakai et al., 1994). According to our CT measurements (−985 to −815 HU), emphysema animals showed attenuation coefficients similar to those observed in other models of elastase-induced pulmonary emphysema (Onclinx et al., 2006; Henriques et al., 2016) and in human emphysema, with lung CT voxels exhibiting attenuation values in the −950 HU range (low-attenuation areas) (Bhavani et al., 2015). A higher predominance of hyperaerated areas increased lung volume, and, after exacerbation, CT values decreased while specific gravity increased as a result of increased alveolar collapse and lung edema.

### Limitations

This study has some limitations. First, no animal model can exactly mimic human COPD exacerbation. However, the model used herein (endotoxin-induced exacerbation of elastase-induced emphysema) is associated with cardiorespiratory and diaphragmatic functional changes which resemble the clinical features of human emphysema. Second, the analysis of extracellular matrix components was focused primarily on collagen and elastic fiber content, and not on organization of other components. Third, the percentage of neutrophils in alveolar septa was not quantified using immunohistochemistry techniques, but rather by visualization by two expert pathologists on slides stained with hematoxylin-eosin. Transmission electron microscopy was also performed to confirm neutrophil infiltration. Fourth, the time course of functional and structural changes in the lung, heart, and diaphragm was limited to 1 day after endotoxin administration; thus, further studies are required to evaluate the aforementioned modifications over a longer period. Fifth, this was not a mechanistic study; it sought to develop and test a new model of emphysema exacerbation that resembles human disease with pulmonary and extrapulmonary compromise.

## Conclusions

In conclusion, this paper reports a model of endotoxin-induced emphysema exacerbation that mimics several pulmonary, cardiovascular, and diaphragmatic features of human disease. This model will allow preclinical testing of novel therapies to improve cardiorespiratory function in COPD, with potential for translation into clinical practice.

## Ethics Statement

This study was approved by the Ethics Committee of the Health Sciences Center (CEUA-CCS 059-15), Federal University of Rio de Janeiro.

## Author Contributions

MO, NN, RS, PP, PS, and PR conceived and designed the experiments. MO, NN, RS, MR, RM, JS, SS, and VC performed the experiments and analyzed the data. MO, PS, and PR coordinated data collection and data quality assurance. MO, NN, SS, PS, PP, and PR wrote the first draft of the manuscript. All authors participated in the manuscript writing process and read and approved the final version.

### Conflict of Interest Statement

The authors declare that the research was conducted in the absence of any commercial or financial relationships that could be construed as a potential conflict of interest.
