# Supplementary material for: Endotoxin-Induced Emphysema Exacerbation: A Novel Model of Chronic Obstructive Pulmonary Disease Exacerbations Causing Cardiopulmonary Impairment and Diaphragm Dysfunction
Source: Front Physiol. 2019 May 28;10:664. doi: 10.3389/fphys.2019.00664 (PMC6546905; doi:10.3389/fphys.2019.00664)
Supplement: Supplementary file 1 [file Data_Sheet_1.PDF]

**SUPPLEMENTARY MATERIAL**

Figure S1. Representative photomicrographs of lung parenchyma stained with hematoxylin-eosin (HE). Original magnification: ×1000. Note increased number of neutrophils in ELA-SAL group (compared to C-SAL) and a further increase after exacerbation (arrows) (ELA-LPS group).

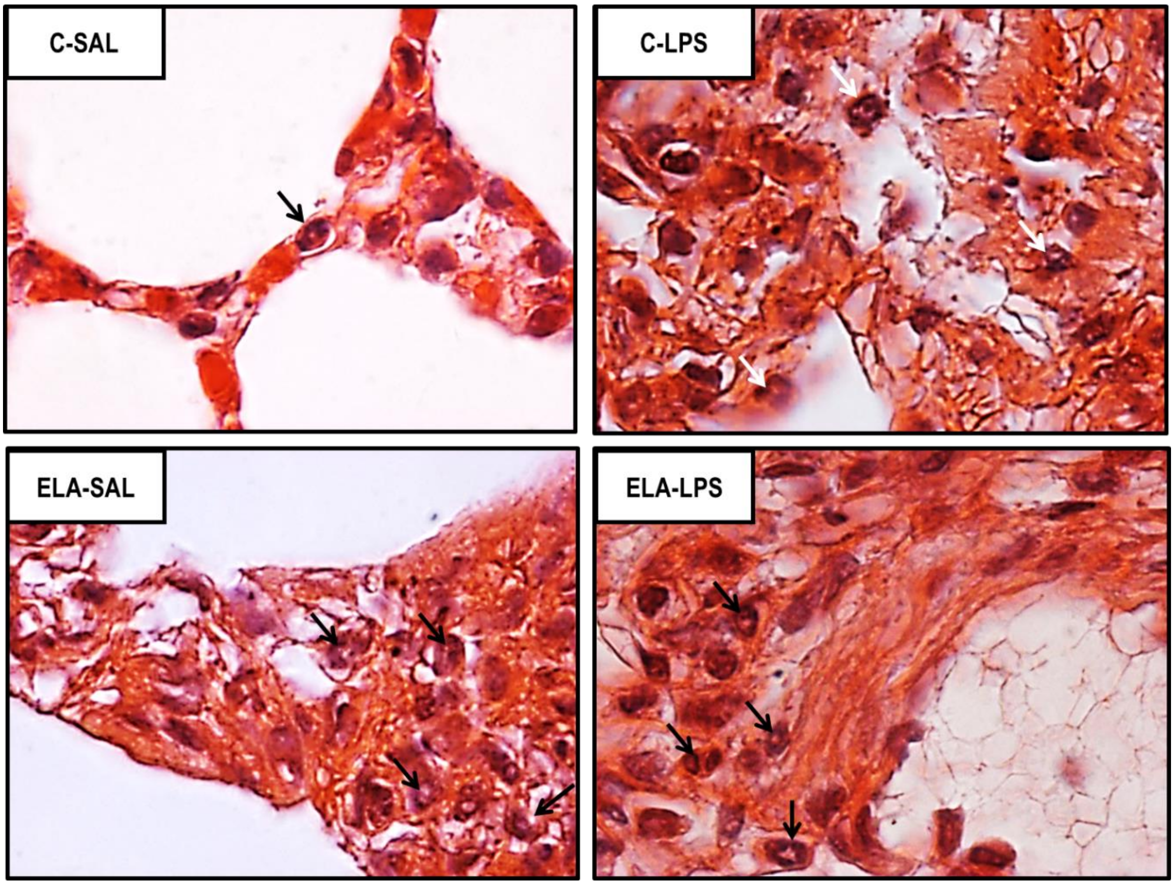

Figure S2: Computed tomography scans (A) and analysis of Hounsfield units (HU), lung volume ($mm^3$), and specific gravity in INITIAL, ELA, and ELA-LPS conditions (B). *Significantly different from INITIAL ($p < 0.05$). #Significantly different from ELA group ($p < 0.05$).

**A**

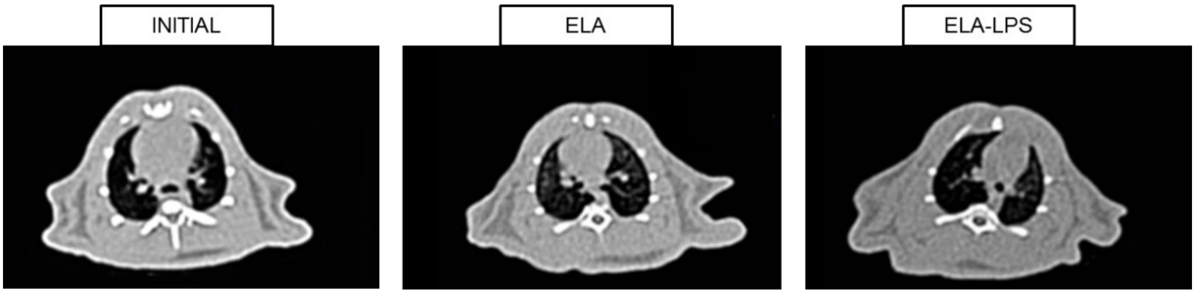


**B**

| | INITIAL | ELA | ELA-LPS |
|---|---|---|---|
| CT numbers (Hounsfield units) | -579.6 ± 69.2 | -921.2 ± 49.9 * | -796.8 ± 58.6 # |
| Lung volume ($mm^3$) | 1758.7 ± 386.2 | 5312.0 ± 451.8 * | 5350.5 ± 754.2 |
| Specific gravity | 0.35 ± 0.03 | 0.49 ± 0.03 * | 0.82 ± 0.10 # |

Figure S3: Representative images of pulmonary blood flow on echocardiography (A) and analysis of PAT/PET, right ventricular end-diastolic area, and diastolic right ventricular wall thickness (B). *Significantly different from INITIAL ($p < 0.05$). #Significantly different from ELA group ($p < 0.05$).

**A**

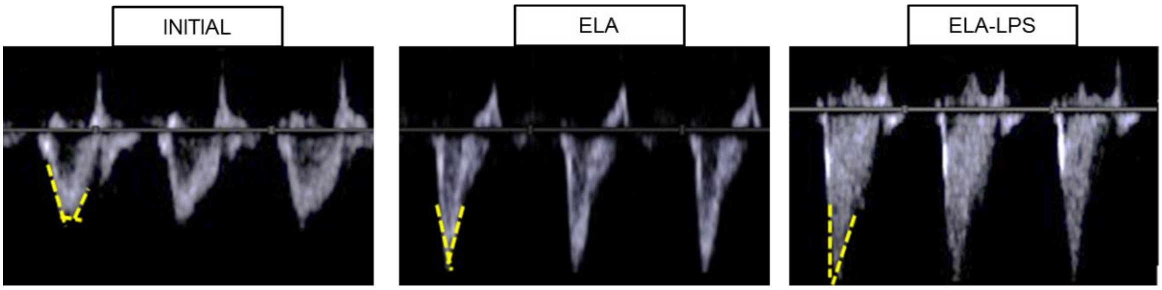


**B**

| | INITIAL | ELA | ELA-LPS |
|---|---|---|---|
| PAT/PET | 0.48 ± 0.03 | 0.34 ± 0.02 * | 0.21 ± 0.04 # |
| Right ventricular end-diastolic area ($cm^2$) | 0.30 ± 0.02 | 0.51 ± 0.02 * | 0.50 ± 0.02 |
| Diastolic right ventricular wall thickness (mm) | 0.05 ± 0.01 | 0.12 ± 0.01 * | 0.15 ± 0.06 |
